# A Treatise on Inflammation

**Published:** 1839-01

**Authors:** 


### PART SECOND.

### Bibliographical Notices.

---

ART. I.—*A Treatise on Inflammation.* By JAMES MACARTNEY, M.D. F.R.S. F.L.S. M.R.I.A. &c.—*London*, 1834. 4to. pp. 214.

IT is with extreme regret that we have been compelled, by want of room, to postpone, for the present, all detailed notice of this most interesting and valuable volume. We had combined Dr. Macartney's with the recent work of Rasori, and with Mr. Palmer's new edition of the immortal treatise of Hunter, in one extensive review of the subject of Inflammation. This we shall lay before our readers in our next Number; but we cannot allow the present to come out without some notice, however imperfect, of Dr. Macartney's work. The station which Dr. Macartney has so long held, with no less credit to himself than advantage to his numerous pupils, as well as his high character as a member of our profession, ensures from us and from all his brethren, respectful attention to anything that comes from his pen, and, in an especial manner, to a work like the present, which professes to embody the opinions and doctrines which he has so long entertained and promulgated as a teacher. But we are confident that he would not desire us to be warped by his authority into an expression of concurrence, where we feel solid reason to dissent; or of approbation, where we find ground for unfavorable criticism. We shall therefore, in our promised article, take leave to express ourselves freely, but impartially, upon the merits of his production, which is to be regarded less as a treatise on inflammation than as an exposition of the author's peculiar opinions on that subject. Those who expect to find in it a comprehensive view of the researches of past and contemporary authors on the various questions discussed, will be disappointed. Scarcely any authority is mentioned but that of Hunter; and he is only brought in when it appeared that his doctrines could be made to accord with those of the author. We do not quarrel with Dr. M. for this; since every man has a right to publish his own doctrines, with as little reference as he thinks proper to those of others; but we think that another title might have been more appropriate than that which Dr. M. has chosen. On many topics, both theoretical and practical, we are disposed fully to agree with Dr. Macartney, and even to attribute to him the credit of having made important improvements in pathological science, and in the therapeutic art. Nevertheless, we are constrained to say that he seems to us very far from having done justice to his own doctrines. He professes, in the preface, to have "sacrificed every quality of the style to perspicuity;" and, though we have little complaint to make of deficiency in the latter, there is an air of poverty and meagerness about the whole exposition that we should not have expected from a teacher of Dr. M.'s experience and acknowledged excellence. We shall not be surprised.

therefore, if the work fails to make as many converts to the author's opinions as he might expect, and even if certain defects in it should prejudice some readers against the really philosophical reasonings and judicious distinctions which we believe it to contain. Some of the plans of treatment recommended in this volume are of the very first importance, and we earnestly recommend our readers to look for them in its pages, without delay. We regret, however, to be compelled to say, that we think the very unnecessarily expensive form in which Dr. Macartney has put forth his book, will be a very serious obstacle to its general circulation. We should certainly wish to see it in the hands of every member of the profession, who is not too much wedded to old opinions and modes of treatment, to resist the introduction of new ones which can be shown to be improvements, both in a scientific and practical view. But the limited means of many of our brethren will not allow them to lay out fifteen shillings on a work which might as well have been sold, in a different form, for five or six. A "rivulet of print running through a meadow of margin" is a very charming thing to the eyes of some who read for the mere gratification of their taste; but it is no refreshment to the eyes of the industrious student, by whose utilitarian soul all the blank space is profanely regarded as so much waste paper.

